# CD46 Protects against Chronic Obstructive Pulmonary Disease

**DOI:** 10.1371/journal.pone.0018785

**Published:** 2011-05-06

**Authors:** Sandra Grumelli, Bao Lu, Leif Peterson, Toshitaka Maeno, Craig Gerard

**Affiliations:** 1 Division of Pulmonary and Critical Care Medicine, Brigham and Women's Hospital, Harvard Medical School, Boston, Massachusetts, United States of America; 2 Centro de Investigación en Medicina Respiratoria, Universidad Católica de Cordoba, Cordoba, Argentina; 3 Department of Medicine Chronic Disease Prevention and Research Program, Baylor College of Medicine, Houston, Texas, United States of America; 4 Pulmonary Division, Children's Hospital, Harvard Medical School, Boston, Massachusetts, United States of America; Rega Institute, University of Leuven, Belgium

## Abstract

**Background:**

Chronic obstructive pulmonary disease and emphysema develops in 15% of ex-smokers despite sustained quitting, while 10% are free of emphysema or severe lung obstruction. The cause of the incapacity of the immune system to clear the inflammation in the first group remains unclear.

**Methods and Findings:**

We searched genes that were protecting ex-smokers without emphysema, using microarrays on portions of human lungs surgically removed; we found that loss of lung function in patients with chronic obstructive pulmonary disease and emphysema was associated with a lower expression of CD46 and verified this finding by qRT-PCR and flow cytometry. Also, there was a significant association among decreased CD46^+^ cells with decreased CD4^+^T cells, apoptosis mediator CD95 and increased CD8^+^T cells that were protecting patients without emphysema or severe chronic obstructive pulmonary disease. CD46 not only regulates the production of T regulatory cells, which suppresses CD8^+^T cell proliferation, but also the complement cascade by degradation of C3b. These results were replicated in the murine smoking model, which showed increased C5a (produced by C3b) that suppressed IL12 mediated bias to T helper 1 cells and elastin co-precipitation with C3b, suggesting that elastin could be presented as an antigen. Thus, using ELISA from elastin peptides, we verified that 43% of the patients with severe early onset of chronic obstructive pulmonary disease tested positive for IgG to elastin in their serum compared to healthy controls.

**Conclusions:**

These data suggest that higher expression of CD46 in the lungs of ex-smoker protects them from emphysema and chronic obstructive pulmonary disease by clearing the inflammation impeding the proliferation of CD8^+^ T cells and necrosis, achieved by production of T regulatory cells and degradation of C3b; restraining the complement cascade favors apoptosis over necrosis, protecting them from autoimmunity and chronic inflammation.

## Introduction

Chronic obstructive pulmonary disease (COPD) is a complex multi variable disease, with raged inflammation triggered mainly by cigarette smoke; regardless of sustained quitting approximately 15% of the ex-smokers develop severe emphysema and COPD. Conversely, 10% of the ex-smokers do not develop emphysema or lung obstruction despite having the same smoking history; the reason for the inability to clear the inflammation in the first group is not well know [1]. The inflammation seen in patients with COPD have an innate and adaptive nature, initially much of the focus of the studies were into the innate response lead by the proteases secreted by macrophages and neutrophils that destroyed the extracellular matrix (ECM) [2]. Later was assessed the presence of CD8^+^ T cells indicating a role for the adaptive response through release of granzymes and perforins that kill epithelial cells [3]. After reports of CXCR3 positive T cells indicating a T helper 1 (Th1) type of immune response [4] we found CXCR3 on macrophages [5] and showed that cross-talk among T cells and macrophages through the binding of interferon gamma (INFγ) inducible protein 10 (IP10), secreted by T cells, to the CXCR3 receptor on macrophages macrophage-metallo-protease 12 (MMP12) was induced, showing a feed forward loop that destroyed the ECM through a non-antigenic cross talk [5]. The reason why this destructive mechanism is not suppressed after the initial smoke injury is removed remains unclear. The hypothesis that a sub-set of ex-smokers with COPD actually has an autoimmune disease to elastin that impairs the inflammation clearance remains debatable [6]–[9], although, there are recent reports of IL17 producing T cells that also associated COPD and autoimmunity [10]–[15]. In normal individuals autoreactive T cells are eliminated in the thymus through a selection of those cells with T cell receptors (TCR) that binds weakly to a self-antigens and may induce anergy. However, there is an intrinsic mechanism that eliminates T cells that strongly binds to self antigens, by up regulation of FAS ligand (FASL) and its receptor CD95 which activating the signaling pathway of caspase (Casp) 8 and proteolysis leads to apoptosis. This is an important regulatory mechanism against autoimmunity because mice and humans with defects in the FAS pathway develop systemic autoimmunity [16]–[18]. When a T cell with an autoreactive TCR that bind strongly or weakly to self antigens has left the thymus there is an extrinsic regulatory mechanism that involves the suppression of these autoreactive T cells by T regulatory cells (Treg) to prevent autoimmunity [17]. Tregs are CD4^+^CD25^+^T cells, they express FOXP3, secrete IL10, suppress cytotoxic (Tc) and helper (Th1) T cells. Tregs are produced by coupling of CD3 with CD46; they are able to regulate the responses adaptive. CD46 not only regulates the Treg production but also the innate response through degradation of the complement 3b protein (C3b), which is required for the cleavage of C5 to C5a, a potent chemoattractant that forms the membrane attack complex (MAC) [19], [21]. There is evidence that Tregs are down regulated in patients with COPD; however, its mechanism remains unknown [6]. The aim of this study was to identify genes that protect ex-smokers from developing end stage COPD/emphysema by clearing the inflammation triggered by cigarette smoke after smoking cessation. We identified CD46 as protective gene that plays a pivotal role between the immune response adaptive and innate; down regulation of CD46 was associated with CD4^+^T cell depletion, CD95 down regulation, and C3b accumulation with increased C5a in the lung. All together these data indicates a key role of CD46 in inflammation clearance and homeostasis of the immune response.

## Methods

### Subjects

This was a multicenter effort to study eighty-seven subjects (Table 1) with and with out COPD and emphysema, which were recruited subsequent written informed consent signature; the pertinent institutional review board for human studies approved all protocols. These subjects were further subdivided in different groups for microarray, flow cytometry or autoimmunity testing as detailed bellow.

**Table 1:** Clinical and Demographic Characteristics of Participants.

	Control(*n = 36*)	Emphysema (*n = 17*)	End-Stage(*n = 34*)
Emphysema a	No	Yes	Yes
GOLD stage	0-1	3-2	4
LVRS b *(n)*			12^f^
Small peripheral Cancer *(n)*	9^f^	17^f^	
Age *(Average ± SD)*	65±12	68±45	60±6
Percent FEV1^c^ *(Average ± SD)*	84±15	63±15	28±6*
Pack-years smoking d *(Average ± SD)*	55±37	60±24	52±17
Ex-smokers *(n)*	23	17	30
QT e *(Years)*	9±10	4±6	11±11

a Determined by Ct scan.

b Lung Volume Reduction Surgery (LVRS).

c Percentage of Forced expiratory volume (FEV1) in one second is *significantly different (p<0.05) for the end-stage group compared to the control group.

d Packs per years (PPY), quitting time (QT) and age values are similar among the three groups.

e Quitting time: continue time without smoking since quitting.

f , The patients had no history of allergy or asthma and had not received oral/systemic or inhaled corticosteroids during the last six months. At the time of study, all patients were free of acute symptoms suggestive of upper or lower respiratory tract infection in the 6 weeks preceding the study.

#### Microarray

Thirteen non-atopic ex-smokers enduring medically necessary lung surgery, due to lung volume reduction (LVRS) or small peripheral carcinoma, as detailed in Table 2, were sequentially entered into the study to investigate their gene expression pattern, after the initial insult has ceased, to assess which genes are protecting ex-smoker without emphysema from developing severe emphysema/obstruction: four subjects of age 78(6), were used as control (control group), which had no to mild obstruction according to their pulmonary function test (PFT), forced expiratory volume in one second (FEV1) percentage of predicted, average (SD), 80(24) and no emphysema as shown by their computed tomography (CT) scan; five patients of age 71(7) with mild to moderate emphysema and COPD 65(15) FEV1% (emphysema group); four patients age 65(5) with severe emphysema and COPD (end-stage group) shown by a FEV1% 34(9) and high resolution computed tomography (HRCT), and/or conventional CT scan performed as previously described [5] was used to classify on the presence or the absence of centrilobular, paraseptal or panlobular emphysema [22]–[24]. COPD was diagnosed according to the criteria recommended by the NIH/WHO workshop summery [25]. All subjects were recruited from the surgical clinic at the Houston Veterans Affair Medical Center. The patients had no history of allergy or asthma and had not received oral/systemic or inhaled corticosteroids during the last six months. At the time of study, all patients were free of acute symptoms suggestive of upper or lower respiratory tract infection in the 6 weeks preceding the study.

**Table 2:** Protective genes.

Microarray Results		qRT-PCR Validation^a^		
Gene ID	Official gene name	Control^b^	Emphysema^b^	End-stage^c^
D84105	Membrane cofactor protein (CD46)	12.0±1.0	9.0±0.5* d	4.0±1*
NM_001228	Caspase 8, apoptosis-related cysteine protease (Casp8)	138.0±45.0	27.0±5‡	34.0±10*
U12767	Nuclear receptor subfamily 4, group A, member 3 (NR4A3)	23.0±9.0	27.0±15.0‡	4.0±1†
NM_001419	Embryonic lethal, abnormal vision, Drosophila Antigen like 1 (Hu antigen R)			
AF074480	Cytidine monophosphate-N-acetylneuraminic acid hydroxylase(CMP-N-Acetylneuraminate monooxygenase) (CMAH)			
NM_005911	Methionine adenosyltransferase II, alpha (MATII)			
BE870509	Hepatocyte growth factor receptor (HGFR)			
AA918442	Insulin-degrading enzyme (IDE)			
NM_006540	Nuclear receptor coactivator 2 (NRCO2)			
NM_003619	Protease, serine, 12 (neurotrypsin, motopsin) (PRSS12)			
M97935	Signal transducer and activator of transcription 1, 91 kDa (STAT1)	23.0±9.0	12.0±1.0	3.5±1.6 †
L38019	Inositol 1,4,5-triphosphate receptor, type 1 (IP3R1)			
NM_021140	Ubiquitously transcribed tetratricopeptide repeat gene, X chrom.(UTX)	1.3±0.4	2.0±1.2	0.6±0.3 §
AF074000	Lectin, galactoside-binding, soluble, 8 (galectin 8)			
NM_005734	Homeodomain interacting protein kinase 3 (HIPK3)			
NM_024530	FOS-like antigen 2 (FOSL2)			
AI638420	Chloride intracellular channel 4 (CLIC4)			
BE963370	BCL2-associated transcription factor 1 (BCLAF1)			

a mRNA expression tested in the same participants by microarray and quantitative RT-PCR which had either ^b^ lung resection for treatment of small peripheral cancer (n = 5); or ^c^ lung volume reduction surgery for emphysema (non-cancer, n = 4).

d Values are expressed as average ± SD, P values are relative to control, calculated using two tailed T students test.

* , p<0.0001;

† , p<0.001;

‡ , p<0.01;

§ , p<0.05.

#### Autoimmunity to elastin

Forty-nine participants were entered into this study, serum from twenty-one individuals were chosen because of their early on set of COPD (EO-COPD) is likely to be caused by an autoimmune disease; they had average age of 50(3) with severe emphysema and end-stage COPD as shown by pulmonary function test (FEV1%) 21(5). Twenty-eight subjects with matching age 49(11) and no obstruction/emphysema according to their FEV1% 95(8) were used as control. All participant had a smoking history of 50(3) pack per year (PPY) for EO-COPD and 21(15) PPY for control, 18 of the EO-COPD and 14 of the control group were ex-smokers all with similar quitting time (QT). Subjects were recruited from the Brigham and Women's Hospital clinic at the Longwood Medical Center, Boston, MA, USA.

#### Lung tissue collection

Tissue from human peripheral lung obtained at the time of the surgery from the furthest away region of the lesion, it was separated in two fractions with the purpose to obtain mRNA for gene expression study and live cells. One fraction was placed in RNA-latter to preserve the tissue for extraction of mRNA using RNeasy kit from QIAGEN. The remaining tissue fraction was used to extract live cells for protein quantification. The extracted mRNA was used for gene expression detection with GeneChip technology and qRT-PCR was performed on the same mRNA samples using validated Assay-On-Demand primers and probes from A&B Applied Biosystems to verify the microarray results on genes of interest on the same patients included for microarray.

#### Gene chip of human lung tissue

RNA was extracted from lung tissue using RNeasy kit from QIAGEN, according to manufacturer's recommended protocol and was used in microarray analysis. We followed established protocols (Affymetrix; the M.D. Anderson Cancer Center microarray core) to synthesize double stranded complimentary DNA (cDNA). Each individual RNA from a total of thirteen subjects were used in Affymetrix Human Genome U133A Array (HG-U133A) [26].

#### Microarrays Data Analysis

Scanned GeneChip DAT files were analyzed by the Gene Chip Analysis Suite Software (Affymetrix) with global scaling to 1,000. Gene screening was performed in Microsoft Excel; first by the p value of the signal for each probe set, 22,284 in total and for each patient. Probes with p values below 0.05 in the 13 patients were considered present and named with a P. Probes with p-value(s) more than 0.05 were considered absent and named with an A. To consider a gene absent within a group it was required that at least 1 patient had p-value(s) more than 0.05 in that particular gene and p-value(s) not more than 0.05 for it to be included within the present group. Genes segregated in this manner were further investigated using Pub Med database.

#### Cell Isolation from surgical lung specimens for Flow cytometry

We used a combination of mechanical fragmentation, enzyme digestion, and centrifugation procedures described previously [22]. Total cell extracted were resuspended to a final working concentration of 1×10^7^ cell/ml in RPMI for use in immune assays and labeled with fluorescent dyes conjugated to anti-CD3, -CD14, CD66, CD46 and CD95 antibodies monoclonal, purchased from BD Biosciences Pharmingen (San Diego, California, United States), for flow cytometry [5].

The patients population was classified as described above for microarray test in control, emphysema and end-stage; we recruited a total 38 patients, their lung function was for controls FEV1% of 80(18) (n = 9), emphysema 63(15) (n = 17), and end-stage 32(6) (n = 12), they had similar age 71(12), 68 (45), 65(8) respectively; and similar smoking history 68(45), 60(24), 54(25) PPY, they were all ex-smoker with similar quitting time 9(10), 4(6), 11(11) for control, emphysema and end-stage respectively.

### Mice

C57 mice were purchased from Jackson Laboratories (Bar Harbor, ME). Age- and sex-matched WT mice were used as controls. The Harvard Standing Committee for Animal Research at Harvard University School of Public Health approved all animal experiments. Transgenic mice deficient in C5aR and CXCR3 were generated in the laboratory of Dr. Gerard.

#### Cigarette Smoke Exposure

WT mice 8–12 weeks of age were exposed to four unfiltered cigarettes per day (University of Kentucky), six days a week for one or four week for acute effect or six months for chronic exposure according to standard procedures [27].

#### Tissue Processing

The *bronchoalveolar lavage* (BAL) was performed by the standard method previously described [28]. The lungs were lavaged four times with 1 ml PBS. Then, the left lung was ligated and removed for western blot analysis. The inflated lungs were fixed in 4% paraformaldehyde, serial mid-sagittal sections in paraffin were obtained for immunohistochemistry and morphometry [29].

#### Immunohistochemistry

analysis of CD46 was done using a custom (rabbit anti-mouse) antibody from Prosci (Poway, CA, USA) at 1:250 dilution. Immunostaining was performed using a Vectastain Elite ABC kit (Vector Laboratories, Burlingame, CA, USA) in which 3,3′-diaminobenzidine (DAB) was the chromogenic substrate. Results are represented as the average count from ten different high-powered fields per slide that have been corrected for mm of alveolar wall.

Immunoprecipitation:. Two mg of whole lung protein homogenate were pre-cleared with 20 µl of protein A (Zymed, San Francisco, CA), centrifuged to eliminate non-specific binding. The supernatant was treated with 20 µl of anti C3b from HyCult Biotechnology (Canton, MA) and incubated for 2 hrs at 4°C. Twenty µl of protein A were added and incubated overnight on a rocker at 4°C. The supernatant was separated by Western blot, performed according to manufacturer instructions. Mouse monoclonal anti-C3b antibody from Hycult Canton, MA was used at 1:200 dilutions and developed with anti-mouse HRP-conjugated antibody (Amersham, Piscataway, NJ, USA). Elastin co-localization was performed stripping the membrane with western re-probe solution and re-probing for elastin with BA4 antibody [30].

#### Peptide ELISA

Costume elastin peptides of 15 amino acids in length (Open Biosystems, Huntsville, AL) were plated at a concentration of 100 µg/ml with carbonate buffer pH 9.4 from a solution kit from BD (OptEIA cat 5505534, San Diego. CA). Human serum, 100 µl diluted 1:100 was used to detect the presence of anti-elastin IgG. Samples were incubated 24 hr at 4°C before washes were performed. Colorimetric measurement was performed using alkaline phosphatase reaction; the optical density was measured at 480 nm.

#### Statistics

Data are expressed as the mean value ± SD unless otherwise indicated. Statistical significance was determined using Mann-Whitney no parametric test, two tails, for human data comparison, and student's t-test (two-tailed distribution with a two-sample equal variance) for animal work.

## Results

### There are 18 protective genes expressed in healthy ex-smokers

From the gene array analysis of ex-smokers with and without emphysema/COPD (Table 1) we identified genetic deficiencies that may underlie the Treg cell deficiency and chronic inflammation in COPD, and/or elastin-specific autoimmunity, focusing our analysis on those genes that were consistently present only in control patients and not in diseased patients; we found 18 gene products that we called protective genes, listed in Table 2, and verified 5 of them by quantitative RT-PCR. Defective expression or polymorphism in these genes (Table 2) may be a predisposing factor for chronic inflammation that leads to COPD.

### CD46 depletion accumulates C3b bond to elastin on lung tissue

**Figure 1:**
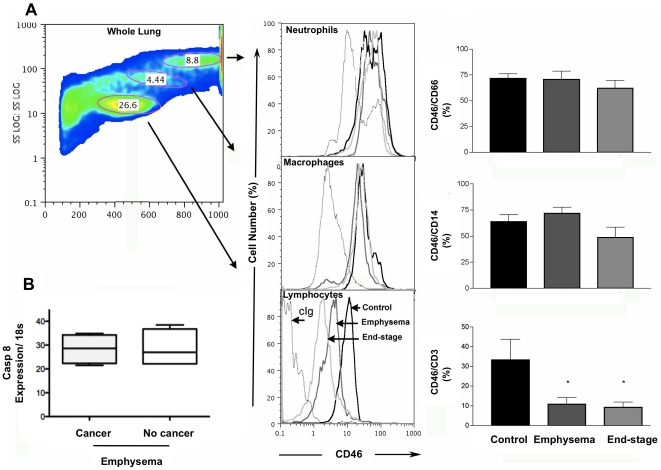
Quantification of CD46 levels. (A) Forward and side scatter plot of lung cells. Circles show the lymphocyte, macrophages and neutrophil populations. To the right a single color histograms showing expression of receptor CD46 from representative control, emphysema and end-stage participants. Pooled data from all participants (control, n = 4; emphysema, n = 4, end-stage n = 5) showing percent (median±SD) of total lung neutrophils, and macrophages expressing CD46; Cumulative values for lymphocytes showed a significative decrease (control, n = 6; emphysema, n = 7, end-stage n = 6, p<0.05) more patient were included with the same lung characteristics. (B) Gene expression of Casp 8, on the same patients, determined by qRT-PCR (median±SD) on emphysema patients with (n = 5) and without cancer (n = 4) shows no different expression level due to cancer away from the emphysemic region (p = 1). Mann-Whitney test was used to determine significant difference.

**Figure 2:**
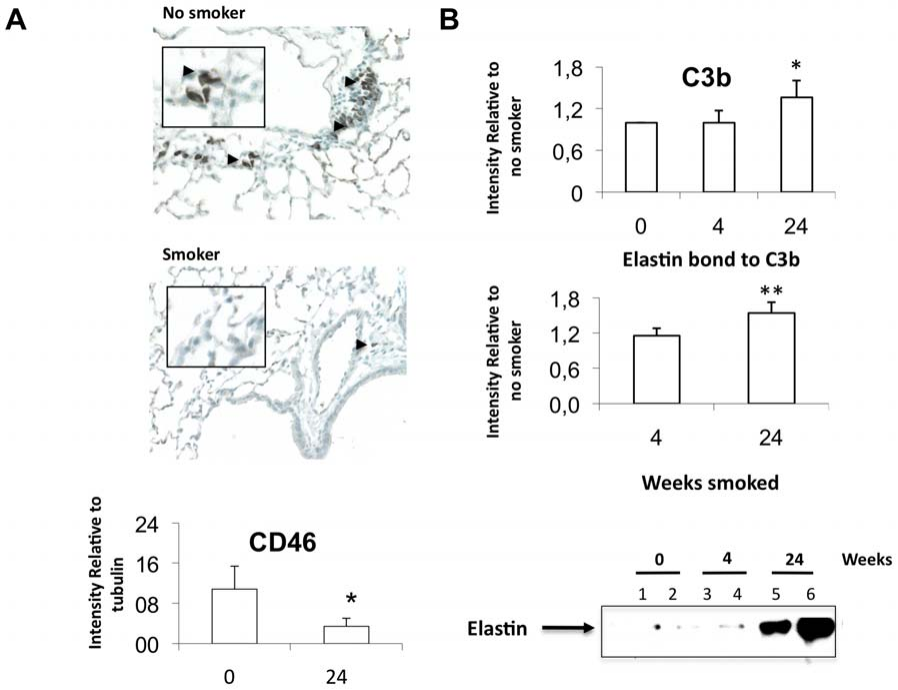
Inflammation in the murine smoking model. (A) Increased expression of CD46 in the lung tissue of no smoker mice compared to smoke exposed mice determined by IHC, arrow heads, and by western western blot on lung tissue homogenates of control mice or smoke-exposed mice (p = 0.02, n = 4, n = 4). (B) Increased deposition of C3b on lung tissue of control mice (n = 3) compared to smoke- exposed mice at 4 and 24 weeks (n = 3) determined by Western blot. Middle plot, immunoprecipitation of C3b from lung homogenate shows a significant increase of C3b deposition upon smoke exposure (p = 0.05). Elastin was stained and detected after stripping the membrane, showing significant increased co-precipitation with C3b (p = 0.01). Student t-test two tails was used to compare both groups; values in the plots represent average ± SD.

The first gene we focused on was ***CD46*** because it plays a pivotal role in the crosstalk between the innate and adaptive immune responses, the over-expression of CD46 in controls was verified at the mRNA level using qRT-PCR and at the protein level by flow cytometry (Table 2). Since T cells were our main interest we added more patients with the same characteristics of the microarray patients (Table 1) to verify differences at the protein level; the results show a significantly higher expression of CD46 on T cells of control patients, without variation on macrophages or neutrophils (Fig. 1A), this data is further supported by the *in vivo* experiments. We also verified that the gene expression differences were due to emphysema and not cancer since control patients had cancer while emphysema patients had cancer or lung volume reduction (LVRS), to that end we segregated the emphysema patients in cancer (n = 5) and no cancer (n = 4) and checked their gene expression level of Casp8 by qRT-PCR to asses that there was not significant differences in the cell survival among those two subset based on the co-founding disease (p = 1, Fig. 1B). Using the smoke-exposed murine model of COPD, we observed a significant decrease in CD46 expression in the lung (Fig. 2A) that was accompanied by significantly increased accumulation of C3b on the lung tissue, which co-precipitated with elastin (Fig. 2B).

### Cigarette smoke activates the complement pathway that suppresses Th1 cells biases

**Figure 3:**
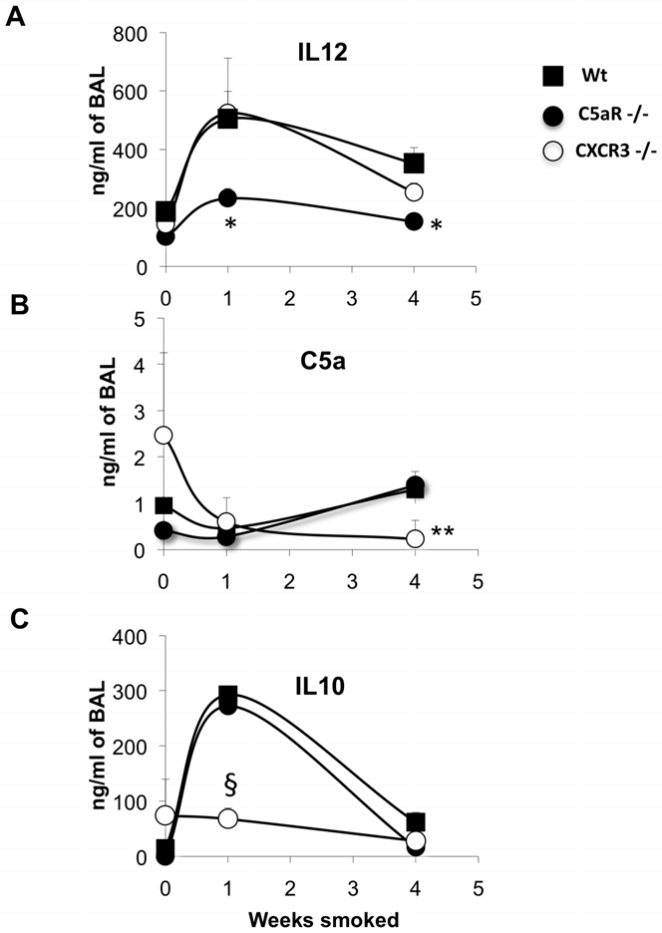
Kinetic of the inflammation in murine smoking model. Secretion of IL-12, C5a and IL-10, measured in mice bronchoalveolar lavage (BAL) of wild type (-▪-Wt), C5aR knockout (-•-C5ar−/−) and CXCR3 knockout (-○-CXCR3−/−) mice exposed at different times to cigarette smoke determined in Wt, C5ar−/− and CXCR3−/−. (A) Top plot show significantly less IL12 (average ± SD) secretion in C5aR −/− (*, p<0.001, n = 3) compared to Wt, while (B) CXCR3−/− shows significantly decrease C5a secretion (**, p = 0.02, n = 3) at 4 weeks smoke and (C) decreased IL10 (§, p = 0.03, and n = 3) relative to Wt (n = 6). P values were calculated using T student test, two tails.

Since C3b is required for the cleavage of C5 into C5a and C5b we quantified the protein down-stream, C5a. Furthermore, the inflammation caused by cigarette smoke (Fig. 3A) in the acute stage (1 week) resulted in a significant increase of IL12 secretion, which biased the immune system to Th1 and was suppressed by C5a up-regulation at the 4^th^ week (Fig. 3B) of inflammation but this response was significantly impeded in C5aR knockout mice at 1 and 4 weeks of cigarette exposure (p = 0.03, n = 3 and n = 5 for Wt) (Fig. 3A). Mice knockout in CXCR3 at the 4^th^ week fails to sustain secretion of both the pro-inflammatory cytokine IL12 (p = 0.06, n = 3) and C5a (Fig. 3D), while C5aR-deficient mice only demonstrate decreased IL12 secretion. After the initial bias to Th1 occurred these cells secrete IP10 that binds to the CXCR3 receptor on macrophages inducing MMP12, and secretion of perforin and granzymes from CD8^+^ T cells that kill epithelial cells [28] releasing large amounts of elastin and causing cell death.

### Decreased expression of CD95 indicates failure to eliminate Tc by Treg

**Figure 4:**
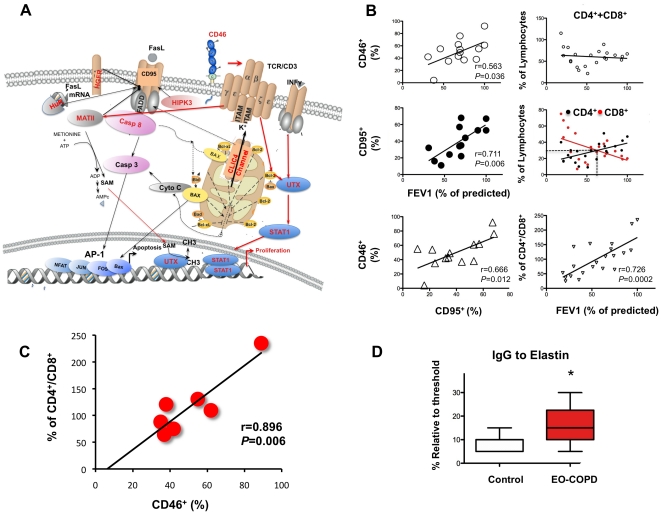
Molecular cross-talk of the protective genes. (A) CD46 coupling with CD3 activates the transcription factor ubiquitously transcribed tetratricopeptide (UTX), which interacts with signal transducer and activator of transcription 1 (STAT1) both translocate to the nucleus to activate proliferation of CD4^+^T cells. T cell receptor activates Bcl-2 that inhibits cytochrome C (Cyto C) released by chloride intracellular channel 4 (CLIC4), which positively regulates Fas Ligand receptor (CD95) mediated activation of Caspase 8 (Casp 8) and apoptosis. Homeodomain interacting protein kinase 3 (HIPK3) phophorilates CD95, and HuR stabilizes CD95 mRNA also positively regulates it. While 2 negative regulators of CD95 are methionine adenosyl transferase II, alpha (MATII) and hepatocyte growth factor receptor (HFGR). Red font signifies protective gene, black arrow apoptosis activation, black bold arrow apoptosis inhibition. Red arrow, proliferation pathway. Red proteins are kinases; blue, transcription factors; light brown, cell surface receptors. (B) Total CD46 expression on the surface of lung cells of patients (n = 14) significantly correlates with their FEV1% in a linear regression using minimum square approximation, with a coefficient r = 0.563 and a goodness of fit p = 0.036. The FasL receptor, CD95, protein expression on the same patients, also, positively correlates with FEV1% with an r = 0.711 and a goodness of fit p = 0.006. There is a significative linear association between cells surface proteins expression, CD46^+^ and CD95^+^ (r = 0.666, p = 0.012). Right top plot, total lung lymphocytes CD4^+^ plus CD8^+^ has a constant number in the lung parenchyma despite their lung FEV1% change with disease progression (r = 0.107, p = 0.6332, n = 22) although there is a significant change in the number of CD4^+^ and CD8^+^ T cells. Right middle plot, in the same patients group, CD4^+^T cells (black circles) correlates directly with the FEV1% (r = 0.549, slope = 0.24, p = 0.008, n = 22), conversely CD8^+^ T cells (red circles) inversely correlates with FEV1% increasing its number with disease progression (r = 0.480, slope  = −0.329, p = 0.024). Bottom right plot shows a direct correlation between FEV1% and depletion of CD4^+^ with increment of CD8^+^ T cells (slope = 8.4, r = 0.726, p = 0.0002). (C) There is a remarkable association among CD46^+^ decrement and CD4^+^/CD8^+^ ratio of T cells, r = 0.896 p = 0.006, n = 7. (D) Increased IgG to elastin in early-onset COPD (EO-COPD) patients, relative to normal controls, tested in n = 21 and n = 28, respectively, plot represents the patients that give signal above the threshold n = 9 for both EO-COPD and control with a significant increased in the diseased group p = 0.038, determined by Mann-Whitney test, values represent median ± SD.

Since memory Treg are created by CD46 coupling with CD3 that suppresses Tc (CD8^+^ T cells), which are eliminated via apoptosis involving Casp8 [31], a protective gene (Table 2), this forms a complex with Fas-associated death domain (FADD) FADD and the FasL receptor (CD95) [32]. In addition, other 5 protective genes regulate CD95 (Fig. 4A), Cytochrome C (Cyto C), released by Chloride intracellular channel 4 (CLIC4), is a positive regulator of CD95 that mediates activation of Casp 8 and apoptosis (Fig. 4A) [33]. Homeodomain interacting protein kinase 3 (HIPK3) phosphorilates CD95 [32] and HuR stabilizes CD95 mRNA [34]. Methionine adenosyltransferase II, alpha (MATII) is activated by proliferation [18] and hepatocyte growth factor receptor (HFGR) sequesters CD95 upon binding to its ligand [35], and both negatively regulates CD95 (Fig. 4A). Thus, we also verified a significant decreased of CD95 that was associated with the CD46 down regulation (Fig. 4B), which has an important role in the down-regulation of immune responses; since severe autoimmunity is developed in human and mice with deficiency in the Fas pathway [16], [18]. CD46 coupling with CD3 activates the transcription factor ubiquitously transcribed tetratricopeptide (UTX) [36] that interacts with signal transducer and activator of transcription 1 (STAT1) [37]–[39] both translocate to the nucleus to activate proliferation (Table 2). T cell receptor activates Bcl-2, which inhibits cytochrome C (Cyto C) [33] and cell death (Fig 4A) [40]. Thus, we inferred the pattern of T cells proliferation with disease progression through the analysis of the total lymphocytes in lung parenchyma; we verified that the increment in CD8^+^ T cells was at expenses of the CD4^+^ T cells population (Fig. 4 B) that directly correlated with the lung function decay (CD4/CD8 vs FEV1, Fig. 4B, bottom right corner). Most importantly, a positive association among CD4^+^ T cell depletion and CD46 reduction was also proven to be significantly linear (r = 0.896, p = 0.006, Fig. 4C) indicating that selective depletion of Tregs with disease progression was associated with down regulation of CD46.

### IgG to elastin is present in serum of patient with early onset of COPD

We thought that if elastin peptides were bound to C3b because they were presented as an auto-antigen, activating T cells, then we should also find IgG to elastin in the serum of patients that manifested early on-set of COPD (EO-COPD), we included for this study a total of 49 participants, 21 EO-COPD and compared them to 28 control, normal subjects. We identified a region of the elastin protein that had more immunogenic properties; the different level of IgG binding to this region among the responders, which were classified as such if their OD was above the threshold set at the highest background, was of (9,9) and no responders (19,12), for control and EO-COPD respectively. EO-COPD group showed a significantly higher (86%) titer of IgG to elastin then normal controls (p = 0.0308 determined by Mann-Whitney test, Fig. 4D). According to these results the sensitivity of the ELISA test used is 43% and the specificity 68%.

## Discussion

In this study we searched for genes that protect ex-smoker from developing severe emphysema and COPD. We found that ex-smokers without emphysema had higher expression of *CD46* in their lungs compared to emphysema/COPD patients, that also occurred *in vivo* in the murine smoking model of COPD where CD46 down regulation was accompanied with accumulation of C3b co-precipitated with elastin and increased C5a which in return suppressed secretion of IL12, from macrophages, stimulated by the cigarette smoke. In contrast, CD46 down regulation was associated with decreased FEV1%, decreased expression of CD95 and CD4^+^ T cell depletion concomitantly with CD8^+^T cells increase in lung parenchyma of emphysema/COPD patients. The proliferation of CD8^+^T may be due to elastin that is being presented as an auto-antigen, since 43% of COPD patients with early onset of the disease had IgG binding to elastin in their serum.

It is remarkable that CD46 depletion rather then ubiquitous was on T cells, and replicated in the murine smoking model which ensures that this effect rather then being mediated by cancer or viral infections is due to cigarette smoke itself, reducing the variables to oxidative stress and free radicals effect on CD46. Free radicals may react in random manner with the cell surface proteins leaving them un-functional or even cleaving them, depletion of this receptor may be underlying the T cell dysregulation and increased cells death associated with cigarette smoke; nevertheless after smoke cessation normal individuals up-regulate CD46 and recover the immunological homeostasis, even some of their lung function. However, the fact that in patients with severe COPD there is persistent down regulation of CD46 mRNA after the noxious irritant has been removed, indicates dysregulation at the transcriptional level, perhaps due to polymorphisms or latent viral infections such as measles or adenovirus [41].

It was expected an accumulation of C3b, in COPD, due to its co-dependence on CD46 for its degradation but the novelty of the co-precipitation of elastin with C3b, seen in humans (data not shown) and mice suggested that elastin might be presented as an antigen. However, the presence of IgG binding to elastin in only 43% of the early onset COPD indicates either the existence of other antigen that may be causing the remaining 57% EO-COPD to develop severe emphysema or, considering the disparity of reports in this matter [6]–[9], it is possible that a abridged sensibility of the tests and/or divergent COPD variants tested may cause dispersion in the results.

T cells ratios vary significantly with the progression of the disease showing an intersection point when the number of CD8^+^T cells reach the same number then CD4^+^T cells (30%) in patients with 60% of FEV1 predicted, from this point on, there is a steep loss of CD4^+^T cells compared to CD8^+^T proliferation; supporting the concept of an extrinsic dysregulation of autoreactives T cells due to loss of Tregs when patients enter in the stages 3 and 4 of GOLD with severe COPD/emphysema [6]. Simultaneously, CD46 down regulation falls bellow 40% suggesting an increased participation of the complement pathway through configuration of MAC and cell lyses. Increased cells lyses is indirectly associated, through CD46, to reduced apoptosis, as shown by the decay of CD95 expression to 30% in patients with 60% of predicted FEV1, reinforcing the hypothesis that these patients will be at risk to develop autoimmunity also due to intrinsic dysregulation in the mechanism responsible for elimination of auto reactive T cells which favors necrosis over apoptosis.

From the kinetic of the inflammatory process seen in the animal model we learn that acute exposure to cigarette smoke initially triggers the Th1 bias inducing IL12 from macrophages [42], which is repressed later by the complement system with the increase of C5a [43], [44]. The early induction of IL12 indicates an effect of cigarette smoke itself on macrophages in the bias towards Th1 which is in agreement with previous reports of INFγ secreting T cells in the lung parenchyma [5], [45]–[47]. The production of IL12 and IL17 are mutually exclusive [48]; this discrepancy with recent reports of IL17 production in human COPD [49] may be because we are using BAL samples from mice, airway and parenchyma, where high amount of IL12 makes it unlikely to have simultaneously IL17 production, unless it is confined to a specific microenvironment of the airways that would account for a small amount of the total cytokines produced in the whole lung, nevertheless, future IL17 testing will shed light on this matter. CXCR3 receptor is thought to be a marker of Th1 bias marker; mice knockout in this receptor show incapacity to mount a full immune response showed by the fact that the secretion of IL12 and C5a are deficiently produced at the 4^th^ week indicating a key role of CXCR3 in disease progression, which turns it an interesting candidate for preventive treatment.

In summary, CD46 plays a protective role against emphysema/COPD by restraining the CD8^+^T cells proliferation and the complement cascade through production of Tregs that regulates the ratios of CD4^+^/CD8^+^ T cells and simultaneously regulating C3b deposition favoring apoptosis over necrosis, thus, protecting this subjects from autoimmunity and chronic inflammation.
